# Evaluation of Optical Coherence Tomography Angiography in Degenerative and Tractional Lamellar Macular Hole

**DOI:** 10.1155/2024/4146294

**Published:** 2024-07-19

**Authors:** Burcu Polat Gültekin, Defne Kalaycı

**Affiliations:** ^1^ Veni Vidi Eye Hospital, İzmir, Türkiye; ^2^ Bilkent City Hospital Department of Ophthalmology, Ankara, Türkiye

## Abstract

**Background:**

This study aims to evaluate the optical coherence tomography angiography (OCTA) findings in cases with degenerative and tractional lamellar macular holes (LMH).

**Methods:**

Two subtypes of LMH cases were included. Seventeen patients had the degenerative subtype, whereas 18 patients had the tractional subtype of LMH. Twenty healthy individuals were enrolled as the control group. The foveal avascular zone (FAZ) and retinal vascular densities in the superficial, deep capillary, and choriocapillary plexuses were analyzed and compared with fellow eyes and healthy controls using OCTA.

**Results:**

The mean FAZ area was wider in the degenerative subtype (0.33 ± 0.14 mm^2^) compared to the tractional subtype (0.24 ± 0.10 mm^2^) (*p*=0.04) and control eyes (0.26 ± 0.10 mm^2^) (*p*=0.03). Foveal vessel densities in the superficial and deep capillary plexuses were lower in the degenerative group than in the tractional group, (21.7 ± 9.8% vs. 26.8 ± 6.9%, *p*=0.01 and 28.5 ± 5.1% vs. 36.9 ± 6.2%, *p*=0.01). Choriocapillary vascular density in the parafoveal area was also lower in degenerative lamellar macular holes compared to the tractional group (60.4 ± 4.7% vs. 63.7 ± 3.9%, *p*=0.03). Compared to control eyes, eyes with degenerative and tractional LMH showed lower vessel densities in the parafoveal and perifoveal areas of the SCP, DCP, and all layers of CC (*p* < 0.05). In the foveal area, the LMH groups showed higher foveal vascular density (FVD) in the SCP than control eyes, while in the DCP, FVD was lower in the degenerative LMH eyes relative to the other groups.

**Conclusion:**

The finding of microvascular changes between degenerative and tractional LMH subtypes highlights their distinct pathologies and supports recent changes in the classification and terminology of this macular condition.

## 1. Introduction

Lamellar macular holes (LMH), first described by Gass as a complication of cystoid macular edema after cataract surgery, are characterized by defects in the inner retinal layers. Optical coherence tomography (OCT) reveals an irregular foveal contour, intraretinal splitting, and the absence of a full-thickness foveal defect with preserved foveal photoreceptors [1–3].

Two distinct types of LMH, tractional and degenerative, have been identified based on structural differences observed in OCT imaging. The tractional type displays schisis-like separation of the neurosensory retina between the outer plexiform and outer nuclear layers, often with an intact ellipsoid layer and associated tractional epiretinal membranes, while the degenerative type features round-edged cavitation, a foveal bump, and lamellar hole-associated epiretinal proliferation (LHEP) [4].

Optical coherence tomography angiography (OCTA), a novel noninvasive technique with high resolution, facilitates the assessment of vascular networks within ocular structures [5, 6]. Recent investigations utilizing OCTA have suggested microvascular alterations in different retinal capillary plexuses associated with lamellar macular holes. However, there is scarce data concerning the distinctions in microvascular structure between degenerative and tractional lamellar macular holes [7, 8].

In this study, our aim was to evaluate the optical coherence tomography angiography (OCTA) findings in the degenerative and tractional subtypes of lamellar macular holes.

## 2. Methods

### 2.1. Study Design

This cross-sectional observational study was conducted at the Retina Department of Ankara City Hospital, and all research and data collection procedures were carried out in compliance with the principles outlined in the Declaration of Helsinki.

### 2.2. Patient Selection

The inclusion criteria comprised individuals aged 18 or older, diagnosed with a lamellar macular hole through biomicroscopy and optical coherence tomography (OCT) examination. Eyes with posterior segment disorders (such as retinal vein occlusion, optic neuropathy, and uveitis), optical media opacities, history of previous laser or vitreoretinal surgery, any intravitreal injection, or any macular pathology observed on OCT were excluded from the study.

Degenerative LMH was defined by the presence of an irregular foveal contour, inner foveal break, and loss of foveal tissue, along with accompanying anatomic features such as epiretinal proliferation, central foveal bump, or disruption of the ellipsoid line. Tractional LMH exhibited an irregular foveal contour, a contractile preretinal membrane, and foveoschisis at the level of the Henle fibre layer.

### 2.3. Clinical Examination

All patients underwent an ophthalmic examination that included an assessment of best-corrected visual acuity (BCVA) and patients with a refractive error ranging from −3.0 to +3.0 spherical equivalent diopters (SE) were included in the study. Anterior segment and fundus examinations, tonometry, and OCTA imaging of the macula were also performed. Twenty age-matched control cases were selected with no significant ophthalmic pathology, normal OCT scans, no history of previous surgical interventions other than uncomplicated cataract surgery, and no systemic diseases affecting retinal vasculature such as diabetes or hypertension.

### 2.4. Image Acquisition

OCTA scans were acquired using the RTVue-XR Avanti system (Optovue, Inc., Fremont, CA) which boasts a high speed of 70,000 axial scans per second and an axial resolution of 5-µm in tissue. This system utilizes the split-spectrum amplitude decorrelation algorithm (SSADA) to provide intrinsic contrast with blood flow. The scanning area covered a 6 × 6 mm region of the macula. AngioAnalytic software was employed to analyze the whole macular region, dividing it into vascular networks of the retina: superficial capillary plexus (SCP), deep capillary plexus (DCP), and choriocapillaris plexus (CC). The software automatically calculated measurements of the foveal avascular zone (FAZ) area and retinal vessel density (VD) from selected retinal layers, defined as the percentage area of vessels on the en-face scans. The foveal, parafoveal, and perifoveal vessel densities (VD) were defined as the vessel density in the foveal region with a diameter of 1 mm, the parafoveal region with diameters ranging from 1 to 3 mm, and the perifoveal region with diameters ranging from 3 to 6 mm, respectively. All scans underwent independent review by two experienced clinicians (BPG and DK) to ensure accurate segmentation. In cases of incorrect segmentation, manual adjustments were made using the AngioVue module of the Optovue software installed in the instrument. Scans with image qualities less than 6/10 and those with motion artifacts were excluded from the study.

### 2.5. Statistics

The OCTA measurements of vessel densities in the superficial, deep, and choriocapillary plexuses, as well as the FAZ areas, were analyzed for both degenerative and tractional subtype LMHs and compared with their fellow eyes and healthy controls.

All variables were tested for normality using the Kolmogorov–Smirnov test in SPSS Statistics software version 20 (IBM Corp., Armonk, NY). Intergroup comparisons for categorical variables were carried out using the Chi-squared test. Differences in FAZ area and vessel densities between LMH subtypes and their fellow eyes were assessed using paired samples *t*-test. Differences between study eyes and control cases were identified using the one-way ANOVA test with Bonferroni correction as a post hoc test. A *p* value less than 0.05 was considered statistically significant.

## 3. Results

### 3.1. Patient Demographics

A total of 35 eyes with LMH and 20 eyes of 20 normally sighted individuals were enrolled in the study. Of the study eyes, 17 eyes were diagnosed with degenerative LMH and the other 18 eyes were diagnosed with tractional LMH.  Table 1 outlines the demographic and clinical parameters of the patients and control eyes. The mean ages were 67.4 ± 8.11 years (range 55–81) for the degenerative group, 71.7 ± 6.97 years (range 60–86) for the tractional group, and 66.1 ± 6.05 years (range 57–77) for the control group, showing no statistically significant difference in age among the groups (*p*=0.85).

In terms of gender distribution, the ratios were 8/9 (female/male) for degenerative LMH, 12/6 for tractional LMH, and 11/9 for control eyes, with no significant difference observed among the groups (*p*=0.67).

Compared to control eyes, those with LMH exhibited significantly worse BCVA (0.25 ± 0.13 logMAR, *p* < 0.01). Additionally, BCVA was worse in the degenerative group (0.61 ± 0.36 logMAR) compared to the tractional LMH group (0.58 ± 0.43 logMAR, *p* < 0.001).

### 3.2. OCTA Parameters

Table 2 summarizes the microvascular characteristics of the LMH groups. In eyes with degenerative LMH, the mean FAZ area was 0.33 ± 0.14 mm^2^, wider than that of fellow eyes (0.26 ± 0.16 mm^2^), though not statistically significant (*p*=0.06).

**Figure 1:**
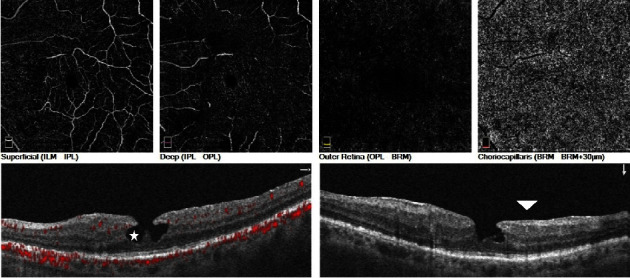
Representative OCTA image of degenerative lamellar macular hole. Intraretinal cavitation (white star) appears as a wide, hyporeflective area involving layers of the neurosensory retina. Lamellar macular hole-associated epiretinal proliferation (white arrow) is observed as homogeneous material with medium reflectivity.

Regarding vascular densities in the superficial capillary plexus (SCP), although no significant difference was found in the foveal area compared to fellow eyes (21.7 ± 9.8% vs. 21.0 ± 9.4%, *p*=0.68), densities were significantly lower in the parafoveal and perifoveal areas of degenerative LMH eyes (40.4 ± 4.9% vs. 42.9 ± 6.1%, *p* < 0.01 and 40.9 ± 4.6% vs. 43.8 ± 5.5%, *p*=0.02). In the deep capillary plexus (DCP), foveal vascular density was lower in degenerative LMH eyes compared to fellow eyes (28.5 ± 5.1% vs. 30.5 ± 3.4%, *p*=0.03), while densities in parafoveal and perifoveal regions were similar (48.9 ± 4.1% vs. 47.8 ± 8.1%, *p*=0.56 and 43.2 ± 5.8% vs. 41.4 ± 6.8%, *p*=0.25, respectively). There was no significant difference between degenerative lamellar holes and fellow eyes regarding vascular densities in the choriocapillary plexus (*p* > 0.05).  Figure 1 illustrates the optical coherence tomography angiography image of a patient with a degenerative lamellar macular hole. In this image, intraretinal cavitation appears as a wide, hyporeflective area involving layers of the neurosensory retina, while lamellar macular hole-associated epiretinal proliferation is visualized as homogeneous material with medium reflectivity.

**Figure 2:**
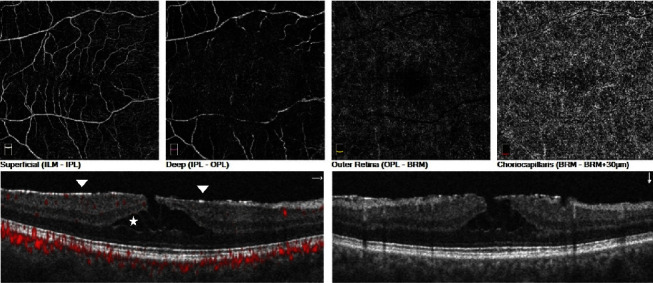
Representative OCTA image of tractional lamellar macular hole. The intraretinal schisis is evident between the outer nuclear and outer plexiform layers (white star). Additionally, an irregular, thin and hyperreflective epiretinal membrane is observed above the inner retinal surface (white arrows).

In eyes with tractional LMH, the mean FAZ areas were 0.24 ± 0.10 mm^2^ and 0.20 ± 0.11 mm^2^ when compared to fellow eyes, with no statistically significant difference found in FAZ area (*p*=0.28). In terms of the superficial capillary plexus, it was noted that the vascular density was significantly higher in the foveal area of eyes with tractional LMH compared to fellow eyes (26.8 ± 6.9% vs. 23.6 ± 8.1%, *p*=0.02). However, no significant difference was observed between the eyes in the parafoveal and perifoveal areas (42.7 ± 5.3% vs. 43.7 ± 6.2%, *p*=0.66 and 42.4 ± 4.5% vs. 43.6 ± 5.4%, *p*=0.50). The vascular parameters in the deep capillary plexus and choriocapillary plexuses were comparable to those in the fellow eyes, with no statistically significant difference.  Figure 2 depicts the optical coherence tomography angiography image of a patient with tractional lamellar macular hole. The intraretinal schisis is visible between the outer nuclear and outer plexiform layers, while an irregular, thin, and hyperreflective epiretinal membrane is observed above the inner retinal surface.

Table 3 presents the comparison of OCTA parameters between degenerative and tractional subtypes with control eyes. In the comparison between degenerative and tractional subtypes, the degenerative group exhibited a significantly wider FAZ area compared to the tractional group (*p*=0.04). The vessel densities in all layers, including SCP, DCP, and CC, were lower in degenerative LMH than in tractional LMH, except for the perifoveal DCP, where vessel densities were slightly higher in degenerative LMH.

Compared to the control eyes, the degenerative subtype exhibited a significantly wider FAZ area (*p*=0.03), whereas the tractional subtype did not show a significant difference (*p*=0.69). Both degenerative and tractional LMH eyes had significantly lower parafoveal and perifoveal vascular densities in the superficial capillary plexus compared to control eyes (*p*=0.01, *p*=0.004). Conversely, foveal vascular density in the SCP of LMH subgroups was higher than in the control group (*p* < 0.001). In the deep capillary layer, the foveal vascular density of the degenerative LMH eyes was significantly lower than that of control eyes (*p* < 0.001), while it was comparable in tractional LMH eyes compared to control eyes (*p*=0.68). Additionally, vascular densities in the choriocapillary plexus were significantly lower in the LMH subgroups than in control eyes (*p* < 0.001).

## 4. Discussion

In the current study, we assessed the microvascular changes in different subtypes of lamellar macular holes and compared them with control eyes using OCTA. Our results indicated that in the degenerative subtype, superficial and deep microvascular changes were more prominent, as evidenced by a larger FAZ area, lower parafoveal and perifoveal SCP vessel densities, and lower foveal DCP vessel densities compared to the fellow eyes. Given that approximately 10–15% of the oxygen supply to photoreceptors is provided by the DCP, the lowest BCVA observed in the degenerative group might be attributed to this condition [9].

Conversely, increased superficial foveal vascularity was observed in the tractional subtype of lamellar hole patients compared to their fellow eyes, suggesting capillary displacement secondary to tractional forces exerted by the epiretinal membrane (ERM) in these eyes. Kashani et al. have previously described the reversible retinal vascular perfusion alteration due to the direct mechanical effect of vitreous traction in these cases [10]. Previous studies have also suggested that tractional forces induce microvascular changes and circulatory disturbances [11].

On the other hand, despite the clinical similarities between degenerative and tractional LMH, our study revealed significant microvascular differences between the two subtypes. The degenerative subtype exhibited a wider FAZ area and lower foveal vascular densities in both the superficial and deep capillary layers. Additionally, the parafoveal choriocapillary vascular density was lower in the degenerative group compared to the tractional group. Dissimilar vascular configuration patterns imply that the tractional forces from ERM in degenerative LMH may vary from those in tractional LMH, indicating that ERM might not have a predominant role in the pathogenesis of degenerative LMH. In the light of these results, the lower vascular densities in degenerative LMH may indicate a slow, chronic, degenerative process that leads to retinal tissue loss and disruption of the ellipsoid zone. This supports the notion that lamellar macular hole subtypes are different clinical entities with distinct etiopathogenesis. As Govetto et al. have noted, the pathophysiological development mechanisms of the degenerative and tractional subtypes of LMH may differ from each other. In the tractional type, there is a schisis-like separation in the outer plexiform and outer nuclear layers secondary to traction, whereas in the degenerative type, there is a chronic separation process involving all layers of the retina [4].

Our study also demonstrated that superficial foveal vascular density was higher in both the degenerative and tractional subtypes compared to control eyes. Additionally, the vascular densities in all SCP, DCP, and CC layers were lower in the LMH subtypes than in the control eyes. The preprint referring to these research findings has been submitted [12]. Pierro et al. reported in their study of 10 cases with tractional ERM-associated lamellar defects that no significant differences were observed in SCP, DCP, and CC layers between LMH and control cases [13]. Another study investigating the differences between degenerative LMHs and healthy eyes described a larger FAZ area (0.39 ± 0.16 mm^2^) and higher vascular density in the SCP compared to healthy eyes, similar to our study [14].

Yeo et al. also observed lower parafoveal vascular densities in SCP and DCP layers in the degenerative subgroup, concluding that tractional and degenerative subgroups have different etiopathogenesis. This aligns with our findings from analyzing the OCTA parameters [8]. In the present study, OCTA parameters of the subgroups were also compared with the fellow eyes, eliminating confounding factors such as systemic disorders, thereby providing deeper insights into the pathogenesis.

In the current study, the vascular densities in the choriocapillary plexuses of the LMH subgroups were significantly lower than those in the healthy eyes. Ahn et al. described comparable choriocapillary flow densities in 19 eyes of LMH and normal controls, in contrast to the significant reduction observed in the full thickness macular hole cases in their study [15].

There are limited data demonstrating the macular capillary plexuses of eyes with LMH in the two subtypes using OCTA. The main limitation of the present study is the relatively small number of cases. Additionally, the variable duration of disease in cases of lamellar macular hole may be a potential factor influencing changes in vascular parameters.

Our results suggest that the macula in two subgroups of LMH shows differences in foveal hemodynamics. The distinct vascular features identified in the two subtypes of LMH imply that they are different clinical entities. This supports the change in nomenclature: the degenerative subtype should be considered as a lamellar macular hole, while the so-called tractional subtype should be renamed as epiretinal membrane-foveoschisis [16].

In conclusion, the significant differences in retinal microvasculature between degenerative and tractional LMHs may shed light on the pathophysiologic mechanisms of their development. Additionally, these parameters may serve as biomarkers for disease progression. These findings support the hypothesis of two distinct pathogenic processes for the different subtypes and reinforce the new classification system. Further research with a larger sample size examining retinal vascular changes would significantly enhance our comprehension of the pathophysiology of lamellar macular holes.

## Figures and Tables

**Table 1:** Demographic and clinical parameters of patients and controls.

Parameters	Degenerative LMH (*n* = 17)	Tractional LMH (*n* = 18)	Controls (*n* = 20)	*p*
Age (mean, years)	67.4 ± 8.11	71.7 ± 6.97	66.1 ± 6.05	0.85^*∗*^
Range (years)	55–81	60–86	57–77	
Gender (F/M) (%)	8/9 (47/53)	12/6 (67/33)	11/9 (55/45)	0.67^*∗∗*^
BCVA (logMAR)	0.61 ± 0.36	0.58 ± 0.43	0.25 ± 0.13	<0.01^*∗*^

BCVA: best-corrected visual acuity, logMAR: logarithm of the minimum angle of resolution, LMH: lamellar macular hole. Data are presented as mean ± standard deviation. ^*∗*^ANOVA test. ^*∗∗*^Chi-squared test.

**Table 2:** Optical coherence tomography angiography parameters of the eyes with degenerative and tractional LMH groups and fellow eyes.

Parameters	Degenerative LMH (*n* = 17)	Fellow eyes	*p*	Tractional LMH (*n* = 18)	Fellow eyes	*p*
FAZ area	0.33 ± 0.14	0.26 ± 0.16	0.06	0.24 ± 0.10	0.20 ± 0.11	0.28
SCP						
Foveal	21.7 ± 9.8	21.0 ± 9.4	0.68	26.8 ± 6.9	23.6 ± 8.1	0.02^*∗*^
Parafoveal	40.4 ± 4.9	42.9 ± 6.1	<0.01^*∗*^	42.7 ± 5.3	43.7 ± 6.2	0.66
Perifoveal	40.9 ± 4.6	43.8 ± 5.5	0.02^*∗*^	42.4 ± 4.5	43.6 ± 5.4	0.50
DCP						
Foveal	28.5 ± 5.1	30.5 ± 3.4	0.03^*∗*^	36.9 ± 6.2	36.3 ± 2.9	0.87
Parafoveal	48.9 ± 4.1	47.8 ± 8.1	0.56	49.9 ± 4.9	50.5 ± 6.5	0.73
Perifoveal	43.2 ± 5.8	41.4 ± 6.8	0.25	43.5 ± 4.8	42.6 ± 6.1	0.64
CC						
Foveal	47.4 ± 6.8	50.8 ± 6.5	0.15	46.6 ± 8.2	47.8 ± 8.9	0.52
Parafoveal	60.4 ± 4.7	61.8 ± 4.7	0.33	63.7 ± 3.9	63.1 ± 4.2	0.13
Perifoveal	62.5 ± 3.2	62.4 ± 3.6	0.53	64.3 ± 3.4	63.0 ± 4.0	0.53

^*∗*^Paired samples *t*-test. FAZ: foveal avascular zone (mm^2^), SCP: superficial capillary plexus, VD (%), DCP: deep capillary plexus, VD (%), CC: choriocapillaris plexus, VD (%).

**Table 3:** Comparison of the OCTA parameters between groups.

	Degenerative (*n* = 17)	Tractional type (*n* = 18)	Control (*n* = 20)	*p* ^1^	*p* ^2^
FAZ area	0.33 ± 0.14	0.24 ± 0.10	0.26 ± 0.10	0.04^*∗*^	0.03^*∗*^
SCP					
Foveal	21.7 ± 9.8	26.8 ± 6.9	18.6 ± 5.70	0.01^*∗*^	<0.001^*∗*^
Parafoveal	40.4 ± 4.9	42.7 ± 5.3	46.6 ± 4.47	0.23	0.01^*∗*^
Perifoveal	40.9 ± 4.6	42.4 ± 4.5	47.1 ± 3.69	0.37	0.004^*∗*^
DCP					
Foveal	28.5 ± 5.1	36.9 ± 6.2	35.4 ± 6.8	0.01^*∗*^	<0.001^*∗*^
Parafoveal	48.9 ± 4.1	49.9 ± 4.9	52.2 ± 4.3	0.56	0.26
Perifoveal	43.2 ± 5.8	43.5 ± 4.8	46.2 ± 5.5	0.86	0.28
CC					
Foveal	47.4 ± 6.8	46.6 ± 8.2	60.9 ± 5.4	0.76	<0.001^*∗*^
Parafoveal	60.4 ± 4.7	63.7 ± 3.9	66.6 ± 3.4	0.03^*∗*^	<0.001^*∗*^
Perifoveal	62.5 ± 3.2	64.3 ± 3.4	68.6 ± 3.2	0.38	<0.001^*∗*^

*p* ^1*∗*^Statistical comparison using independent samples *t*-test between LMH subtypes. *p*^2*∗*^One-way ANOVA test between groups. FAZ: foveal avascular zone (mm^2^), SCP: superficial capillary plexus, VD (%), DCP: deep capillary plexus, VD (%), CC: choriocapillaris plexus, VD (%).

## Data Availability

The data used in this study can be obtained from the corresponding author upon reasonable request.
